# MorphoCloud: Democratizing Access to High-Performance Computing for Morphological Data Analysis

**DOI:** 10.12688/f1000research.176328.1

**Published:** 2026-01-14

**Authors:** A. Murat Maga, Jean-Christophe Fillion-Robin

**Affiliations:** 1 Pediatrics, University of Washington, Seattle, Washington, 98152, USA; 2 Center for Developmental Biology and Regenerative Medicine, Seattle Children's Research Institute, Seattle, Washington, 98101, USA; 3 Kitware Inc, Clifton Park, NY, 12065, USA

**Keywords:** 3D digital morphology, cloud computing, 3D visualization, morphometrics, image analysis

## Abstract

**Background:**

The digitization of biological specimens has revolutionized morphology, generating massive 3D datasets such as microCT scans. While open-source platforms like 3D Slicer and SlicerMorph have democratized access to advanced visualization and analysis software, a significant “compute gap” persists. Processing high-resolution 3D data requires high-end GPUs and substantial RAM, resources that are frequently unavailable at Primarily Undergraduate Institutions (PUIs) and other educational settings. This “digital divide” prevents many researchers and students from utilizing the very data and software that have been made open to them.

**Methods:**

We present MorphoCloud, a platform designed to bridge this hardware barrier by providing on-demand, research-grade computing environments via a web browser. MorphoCloud utilizes an “IssuesOps” architecture, where users manage their remote workstations entirely through GitHub Issues using natural-language commands (e.g., /create, /unshelve). The technology stack leverages GitHub Issues and Actions for front-end and orchestration respectively, JetStream2 for backend compute, and Apache Guacamole to deliver a high-performance, GPU-accelerated desktop experience to any modern browser.

**Results:**

The platform enables a streamlined lifecycle for remote instances, which come pre-configured with the SlicerMorph ecosystem, R/RStudio, and AI-assisted segmentation tools like NNInteractive and MEMOs. Users have access to a persistent storage volume that is decoupled from the instance. For educational purposes, MorphoCloud supports “Workshop” instances that allow for bulk provisioning and stay online continuously for short-term events. This identical environment ensures that instructors can conduct complex 3D workflows without the typical troubleshooting delays caused by heterogeneous student hardware.

**Conclusion:**

MorphoCloud demonstrates that true scientific accessibility requires not just open data and software, but also open infrastructure. By abstracting the complexities of cloud administration into a simple, command-driven interface, MorphoCloud empowers researchers at under-resourced institutions to engage in high-performance morphological analysis and AI-assisted segmentation.

## Introduction

### The harnessing 3D data in biological sciences

The last decade has witnessed a paradigm shift in biological data collection. Over the years, the NSF Biology Directorate has made significant investments in the 3D digitization of biological specimens held in natural history collections. Initiatives like oVert ^1^ (OpenVertebrates) and MorphoSource ^2^ have generated and published hundreds of terabytes of high-resolution 3D data, ranging from surface models to high resolution Computed Tomography (CT) scans. These projects have successfully democratized *access to data*, ensuring that a researcher in a remote location or a rural institution can download the same digital specimens as a curator at a major natural history museum.

### Bridging the software gap: SlicerMorph

While the digitization of specimens removed physical barriers, a significant “software barrier” remained. Historically, interacting with 3D biological data required expensive, proprietary visualization software. Furthermore, the workflows were fragmentary, necessitating serial use of 4-5 different software packages to accomplish the tasks of importing data from scanners, visualizing, segmenting and analyzing them. This fragmented workflow with incompatible formats and proprietary software created significant data management challenges, as the same specimen was being represented in 4-5 different formats and data representation in various stages of the pipeline. These formats were incompatible with each other, making going back and forth through the workflow to change and improve very difficult, if not impossible.

To overcome these challenges we devised the SlicerMorph project. ^3^ 3D Slicer (aka Slicer) was chosen as the primary platform to develop the extension because Slicer was already a very capable application, providing in a single platform visualization, processing and analysis of 3D volumetric data. ^4, 5^ Missing functionality could be added on thanks to its open-source and modular nature. SlicerMorph extended the existing core functionality of Slicer with generic (ImageStack) and vendor specific (SkyscanReconImport, GEVolImport) functionality to import imagestacks from microCT and other modalities; implemented the Generalized Procrustes Analysis (GPA) for visualizing shape variation and other geometric morphometrics modules (e.g., PseudoLMGenerator, PlaceGridLandmarks, ProjectSemiLMs), as well as fully automated landmarking tools like ALPACA and MALPACA. ^6– 9^ Within in the SlicerMorph extension, there are additional modules that provide convenience functions such as segmenting endocranial space in mammalian skulls (SegmentEndocranium), alignment and registration of 3D models (FastModelAlign), acquiring high-resolution 3D renderings (HiResScreenCapture). SlicerMorph team continues to work with the other Slicer developers to incorporate changes into the core functionality of the Slicer, such as the new markups infrastructure that enabled landmarking functionality, help diagnose and resolve issues regarding using and interacting large datasets in Slicer including rendering, segmentation and saving, as well as adding new functionalities to (e.g., ColorizeVolume). At this juncture, all fundamental digital morphology tasks such as importing, visualizing, segmenting, and analyzing can be done completely within Slicer. For domain specific inferential statistics on morphometric data, SlicerMorphR R library provides convenient import of SlicerMorph’s GPA results into the R ecosystem with a single line of code.

At the same time SlicerMorph project has grown from a single extension to an ecosystem of integrated extensions that work together for complex workflows. For example, now SlicerMorph’s Photogrammetry extension ^10, 11^ allows converting large number of photographs into 3D textured models, which can then be landmarked using the existing tools in Slicer, which in return can be fed into SlicerMorph’s Dense Correspondence Analysis (DeCA) toolkit that simultaneously create 3D template of models and sample dense semi-landmarks, ^12, 13^ and finally the new output can be can be analyzed with the GPA module in the SlicerMorph extension proper. Mouse Multi Organ Segmentation (MEMOs) extension uses deep-learning to rapidly segment 50 structures diceCT scans of mouse embryos. ^14^ Segments generated by MEMOs can be immediately fine-tuned with the Slicer’s Segment Editor. ScriptEditor extension allows taking example python codes from Slicer’s Script Repository and tweaking it for the user’s specific needs all inside the Slicer’s environment with support for code auto complete and function discovery and help documentation. The emerging MorphoDepot extension allows teams, whether they are geographically separate collaborators, or instructor and students in a class, to work jointly on the same segmentation using the well-established fork-and-contribute model of open-source development.

All in all, it is now possible to conduct all digital morphology tasks entirely within the Slicer platform, and maintain full control over the workflow. Any unique functionality that is currently not available can be implemented rapidly, thanks to the growing number of AI-assisted coding platforms that are trained on the fully open Slicer source code, documentation and its Application Programming Interface (API).

### The compute gap

Despite the availability of open data and open software, a final barrier prevents the full democratization of this science: the *hardware* gap. While the application can be free, the computational cost of running it is not. Processing high-resolution microCT scans requires workstations with high-end GPUs and large amounts of RAM. Particularly for segmentation a typical rule of thumb is 4-8 times more physical memory than the size of the dataset. A “typical” high-resolution microCT scan can easily be 2048 voxels (or more) in each dimension. If the intensities in this image are represented by 16-bit data, it means simply importing this data as a 3D array would require 16GB of physical memory. During the segmentation, necessities of having multiple copies of this array mean transient memory usage can be as large as 60-100GB, which often exceeds resources on personal computing devices. This large resource requirement, coupled with the need to have data-center grade GPUs to benefit from emerging AI technologies to process and segment large microCT datasets creates a “digital divide.” Research-intensive universities often have high performance computing environments, which can be leveraged to accomplish these tasks by their faculty and students. Also, often there are shared computer labs with relatively modern and powerful workstations, since computationally heavy courses are fairly common in such institutions. On the other hand, Primarily Undergraduate Institutions (PUIs) and other teaching focused institutions may lack such resources. An instructor who wants to incorporate existing 3D morphological data to supplement their curriculum, not only tasked with identifying which datasets to use, but where and how students will access these resources.

### The MorphoCloud mission

ACCESS is a program established and funded by the U.S. National Science Foundation to help researchers and educators, with or without supporting grants, to utilize the nation’s advanced computing systems and services, at no cost. ^15^ However, most of the resources within the ACCESS program are traditional high-performance computing environments that are set to be used as queue-based batch work systems and are not conducive to the interactive computing that is necessary for digital morphology. Alternative to these HPC systems are cloud farms that provide virtual machines that come in many different configurations and can be “rented” on demand. While cloud farms may offer cost-effective solutions to “rent” powerful computers without investing significant sums of capital equipment, they have their own challenges. These virtual computers need to be configured from the bare operating system, meaning the “tenant” needs to configure the network, install all the software they need and maintain it securely. The second challenge is, commercial cloud farms charge rent by the minute the virtual computer is online, regardless of if the computer is actively being used or simply sitting idle. So, any computer that is not being used needs to be turned off and “shelved” to avoid charges. Any “run-away” or “stray” virtual machine has the potential to rack up charges in thousands of dollars (or exhaust the educational credits) until it is discovered and turned off. While there are orchestration tools and technologies to avoid such situations, these are jobs for full-time system administrators, which are hard to come by at most PIUs and other non-research focused academic institutions, and are not tasks most morphologists or researchers in related fields are familiar with or have the bandwidth to acquire the skills.

JetStream2 is a public cloud farm that is part of the ACCESS program funded by NSF. ^16^ Like any resource in the ACCESS program, any US researcher can request allocation on the JetStream2 at no cost. However, they still need to accept the responsibility to provision their own instances with the software, and keep tabs on their running instance not to exhaust their resources. So, the challenges associated with commercial cloud vendors still apply to JetStream, only the cost aspect of it is changed.

Here we introduce the MorphoCloud, an “IssuesOp” based platform built on the JetStream2 and allows users to provision and manage powerful remote workstations (aka instances) with simple commands. ^17^ Our aim is to bridge the compute gap by providing “one-click” access to modern, research-grade computing infrastructure without requiring users to become system administrators. Created instances can be accessible via web browser and already come with the Slicer and SlicerMorph ecosystem preinstalled, enabling research and teaching activities to commence at the very moment the instance is online.

## Methods

The core design philosophy of MorphoCloud is “IssuesOps”—a command-driven interface. MorphoCloud uses the Github Issues as the primary means to request, get approved and control the instances. Users interact with the system by typing natural-looking commands (e.g., /create, /unshelve, /renew) as comments on an issue. This approach provides a simple, collaborative, and persistent record of all interactions. There are only two requirements to use MorphoCloud instances: A public ORCID, and a Github account, both of which are free to acquire.

### Overview of MorphoCloud technology stack

MorphoCloud uses existing, established open-source projects to facilitate the functionality. These are:

• **Frontend** (User Interface): GitHub Issues. The issue tracker serves as the dashboard, where users request resources and receive status updates via bot comments.
• **Orchestration**: GitHub Actions. These workflows parse user comments, validate permissions, and execute the necessary logic. Crucially, the workflows run on a Self-Hosted Runner that resides within the JetStream2 cloud network, allowing direct, secure communication with the OpenStack API.
• **Backend** (Compute): OpenStack running on JetStream2. This provides the raw virtualization layer.
• **Provisioning**: Cloud-Init and Ansible. When an instance is spawned, a custom user-data script bootstraps the environment. It installs Ansible in a virtual environment and pulls a specific configuration playbook from the Exosphere repository ^18^ customized for the MorphoCloud to set up the remote desktop environment.
• **Delivery**: Apache Guacamole. A clientless remote desktop gateway that renders the desktop environment to HTML5. This allows users to access their high-performance instance via any modern web browser, bypassing local hardware limitations.

### Security model

Security is managed through a “vendorized” workflow approach. The complex logic resides in a central repository (MorphoCloudWorkflow), while user projects (eg. MorphoCloudInstances) import these workflows. Sensitive credentials (SSH keys, OpenStack API tokens) are stored as GitHub Repository Secrets, never exposed in the issue comments, and only accessible to repository owners. Access control to the managed instances is enforced via a defined list of administrators who must approve instance creation and management requests using the/approve command.

This flexible design allows anyone who obtains their own allocation on the JetStream2 to fork the MorphoCloudWorkflow repository, customize it for their own use cases (e.g., different software tools and/or user interface) and deploy it for their own community. Step-by-step instructions for creating custom deployments similar to MorphoCloud can be found on the MorphoCloudWorkflow repository. ^19^

## Results

**Figure 1.**
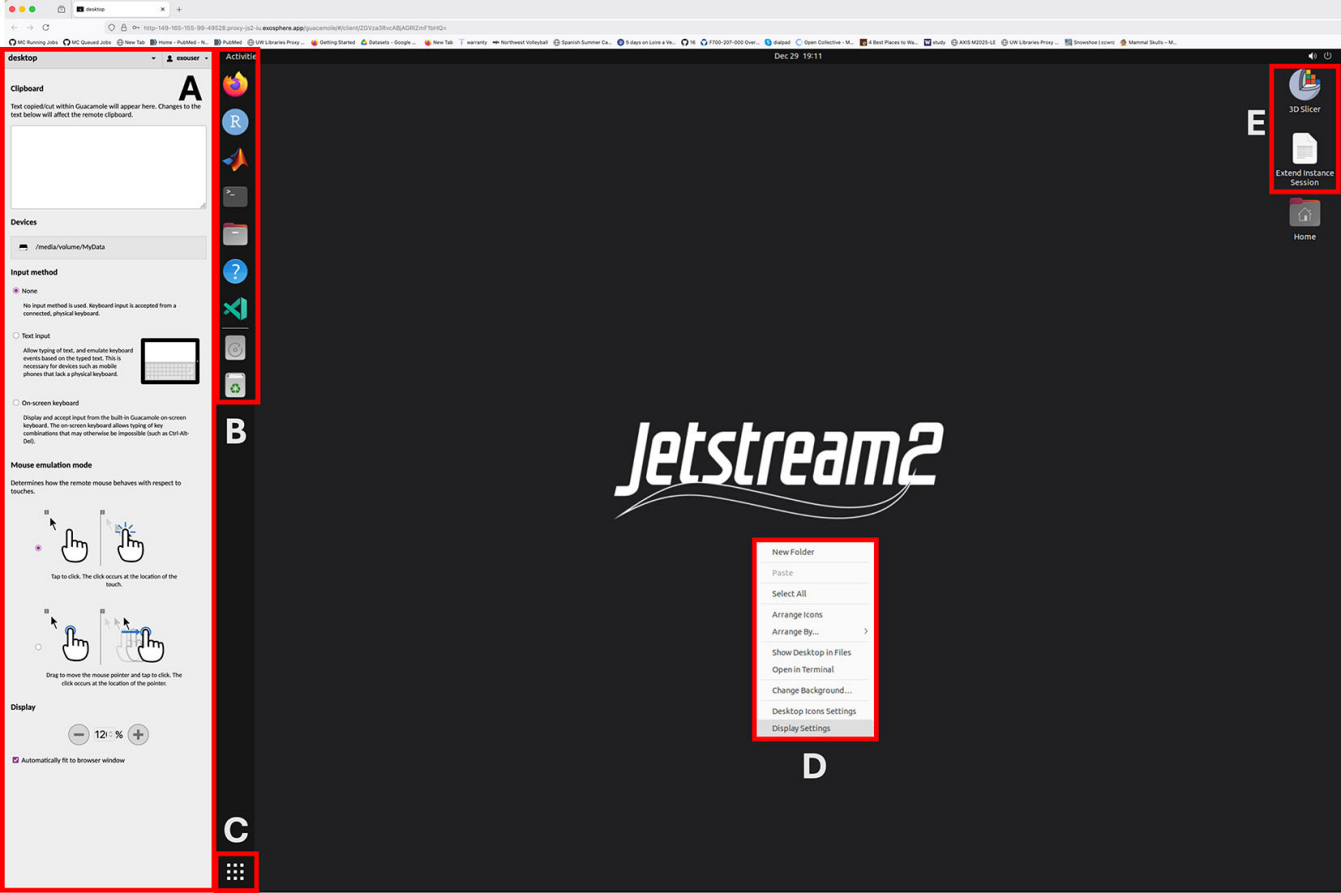
Apache Guacamole (Web browser access) interface. **A:** Side panel allows copy/paste text into the remote session, browse and download files on the remote server and adjust screen zoom levels. **B:** Shortcuts to commonly used applications and to **MyData** storage volume. **C:** Displays list of available applications (searchable) **D:** Right mouse click brings desktop context menu, including changing screen resolution and other visual desktop settings. **E:** Icons for extending the current session for another 4 hours and the 3D Slicer applications. Users can extend their session as many times as they want from this shortcut.

This is the lifecycle of a MorphoCloud instance from an individual user’s perspective:

1. **Request and Approval:** User opens a new Issue using a standardized template identifying themselves via their ORCID profile, briefly explaining their project, and choosing an instance type ( Table 1). The MorphoCloud team reviews the request to make sure the request fits in the MorphoCloud intended usage and approves it often within a few hours. If there are concerns or clarifications needed, more comments are added to the user’s specific github issue.
2. **Instance Initialization**: An approved user can initialize the process by issuing /create, command, which starts provisioning the instance with all the necessary software, along with the user’s persistent storage volume. The issue page displays each provisioning stage so that the user can see the progress (or notice errors). The same page is where all the commands are issues to manage the instance ( Table 2).
3. **Notification**: Once an instance is ready and online, the system indicates that provisioning is completed, and sends the user an email with access credentials.
4. **Access to remote desktop**: User can access the instance via a web browser by clicking the access URL and entering the provided credentials ( Figure 1). Alternatively, the user can connect to the instance via the VNC protocol for a slightly more performant and more native-like experience. Command line access to the instance via SSH protocol is also a possibility.
5. **Shelving and Unshelving:** An active instance stays online up to four hours, unless the user chooses to further extend their session. Users can extend their sessions four hours at a time by clicking an icon on the remote desktop. An inactive remote desktop session will “shelve” itself automatically at the end of the last four-hour extension. Alternatively, when done, the user can shelve their own instance by typing the command /shelve on their issue’s page. A shelved instance can be made online again by entering the /unshelve command on the issue page. Unshelving is much faster than fully provisioning the instance and often the instance is online within a few minutes after the command is entered. A new email with access credentials is sent to the user each time as those may change after shelving and unshelving. If the credential email is not received or lost for any reason, it can be re-requested via/email command.
6. **Instance expiration and renewal:** Each instance has a life span of 60 days, after which the instance expires and gets deleted automatically. 60-day countdown starts from the time /create command is issued by the user. As the life span is nearing, a remainder email is sent to the user about the upcoming expiration. The user then can issue the /renew command to extend the life span for another 60 days. If the user takes no action, both the instance and their persistent storage volume will be deleted at the end of the 60 days.
7. **Additional commands for troubleshooting:** Due to reasons beyond our control (e.g., networking lost during provisioning, faulty host or GPU), instances may be left in an inaccessible state. In such cases, additional commands such as /delete_instance can be issued by the user to purge the instance and reprovision their own instance by reissuing the/create command. This is often the simplest and fastest solution to regaining access and is harmless since this command only removes the instance and the user’s persistent storage volume is untouched.

**Table 1.** Available instance types, specifications and use cases. g3.l is the default instance type on the MorphoCloud.

Flavor	RAM	Cores	GPU	Storage	Use cases
g3.l*	60GB	16	A100 (20GB)	100GB	General purpose morphology and morphometrics
g3.xl	125GB	32	A100 (40GB)	100GB	Photogrammetry, NNInteractive, AI applications
m3.xl	250GB	64	None	100GB	Computationally intense tasks that don’t require GPU; eg., Image registration with ANTsPy
r3.l	500GB	64	None	100GB	Image registration with larger dataset
r3.xl	1000GB	128	None	100GB	Image registration with larger dataset

**Table 2.** List of most commonly used commands as “IssuesOps”.

Command	Function
/create	Provision a new instance (one time only, unless its deleted)
/shelve	Suspend (turn off ) instance (after 4h, instance auto-shelves)
/unshelve	Bring a shelved instance online again
/renew	Extend the instance by another 60 days (one time only)
/email	Resend access email (valid only if the instance is online)
/delete_instance	Delete an instance (only used as a troubleshooting step)

To support instructors offering short courses and workshops centered on the SlicerMorph ecosystem, MorphoCloud also includes a “Workshop” instance request type. The primary purpose of the workshop instance type is to bulk provision many instances and distribute the access credentials to participants prior to the event. Moreover, these instances do not automatically shelve themselves after four hours of inactivity, but remain online continuously. However, their life span is much shorter. Workshop instances can only be requested for up to five days (plus 24h of grace period for setup). The course organizer makes the request on behalf of participants, receives the access credential emails for all instances created for the event, and is responsible for distributing them to the participants.

### Data management

As briefly mentioned above, the instances and where the user data is stored is decoupled by design. Instances are considered “ephemeral” and as such can be deleted and recreated to troubleshoot or to upgrade the base software tools deployed. Therefore, we advise the MorphoCloud users not to store any critical data on the boot disk of the instance, but instead store everything in their private, persistent storage volume. This 100GB volume is mapped as an external disk under the /media/volume/MyData. Primary document root for Slicer and other applications also points to this folder.

Apache Guacamole interface (aka the web browser connection) provides a convenient utility to traverse the remote file system and to download individual files by double clicking them ( Figure 1). Similarly, while using the Guacamole interface, if the user drags and drop a file from their own local computer to the remote desktop, the file will be automatically uploaded to the remote computer and will be placed under the persistent storage. For bulk data ingress and egress onto the instance using standard SFTP and SCP protocols is suggested.

## Use cases

**Figure 2.**
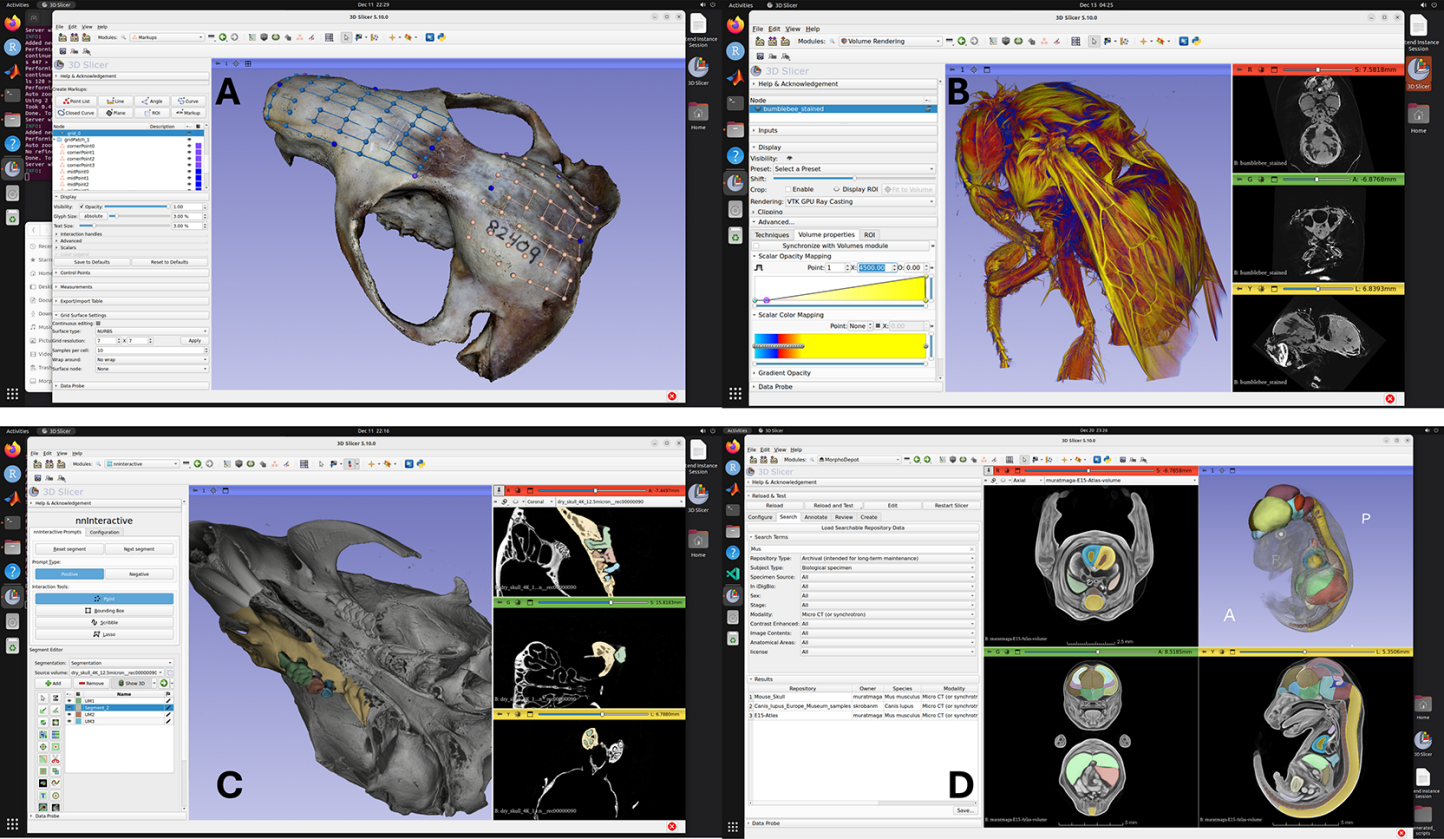
Screenshots of MorphoCloud instances running different Slicer extensions. **A.** Rendering of textured 3D model reconstructed through SlicerMorph Photogrammetry extension, with landmarks created via PlaceLandmarkGrid module in SlicerMorph extension. **B.** 3D Volume rendering of a stained bumblebee on a g3.xl instance. Dataset is 1824x1836x2023 voxels (16-bit) and is 12.5GB in size. **C.** Interactive segmentation of molar tooth row as separate segments using NNInteractive extension. User prompts the tool for the regions to keep and regions to remove in a segment. The result shown required 8 prompts (clicks) and took less than 2 minutes to generate. **D.** MorphoDepot collaborative segmentation framework (displaying search results).

MorphoCloud is designed primarily to be used by people who need intermittent access to powerful computers to process large 3D datasets interactively and cannot justify investing significant capital expense for sporadic usage of a technology that gets depreciated and outdated quickly ( Figure 2A- 2B).

MorphoCloud is also particularly useful is intense short courses and workshops focused on digital morphology and related fields, as it isolates a lot of the technological complexity associated with running such events. Traditionally in such events participants are expected to bring their own computing devices. Undoubtedly these computers are a heterogeneous mixture of operating systems, hardware configurations and restrictions (e.g., devices centrally managed by IT departments). The time spent on troubleshooting one-off challenges faced by the participants can easily derail the schedule. Because MorphoCloud provides an identical environment to all users, once instructors run their workflow on their instances, they can be confident that it will work for everyone as intended. For such events, instructors can provision all the nodes the day before the activity, distribute the access credentials to the user on the day of the event, and because the only software requirement from the user’s side is a web browser, the event can proceed in a timely fashion. Because workshop instances do not auto-shelve themselves in time of activity, once they are provisioned, they are guaranteed to stay online throughout the event. However, again there is no procedure guarantee sufficient resources will be available at the time of provisioning. That is why the workshops are given a 24h grace period to launch their instances. In our experience the default g3.l flavor of MorphoCloud is often consistently available for events up to 35 participants ( Table 1). Another issue that such event organizers must be mindful of is that JetStream2 engineering team have scheduled and (occasionally) emergency maintenance of the system. Depending on the type of the maintenance the system might be partially or completely unavailable to the users. We advise checking the JS2’s planned outage calendar, or getting in touch with the admins, before planning such an event.

Another use case MorphoCloud proved to be very convenient is using AI-assisted technologies in digital morphology. While pre-trained deep-learning networks—ranging from fully automated pipelines to interactive, promptable models ( Figure 2C)— offer transformative potential for processing CT data, their practical implementation is hindered by a “multi-layered cake” of software dependencies. The deployment of open-source AI tools requires a precise alignment of several distinct architectural layers. At the foundation, the hardware-driver interface (e.g., NVIDIA CUDA) must be exactly compatible with the computational frameworks (such as PyTorch or TensorFlow). Above this, a complex lattice of mathematical and image-processing libraries must be version-matched to the specific segmentation tool. As such, just obtaining the right environment to test a new AI tool can itself be a large time sink. MorphoCloud mitigates these complexities by providing the correct and current software stack that is tested with the Slicer. By hosting these “layered cakes” in a pre-configured, cloud-based infrastructure, often all the user needs to do is to install the extension from the Slicer’s catalogue, and test its functionality in a few minutes.

Finally, MorphoCloud instances allows quick start with the collaborative segmentation framework MorphoDepot. MorphoDepot is a distributed, github based platform to share and collaborate on segmentation using open-source 3D Slicer biomedical image computing platform. ^20^ Its primary goal is to use github infrastructure to manage multi-person segmentation projects, particularly in context of classroom assignments. A repository is used to manage segmentation of a specimen (e.g. a microCT of a fish) and issues are assigned to people to work on parts of the segmentation. Pull requests are used to manage review and integration of segmentation tasks. The Slicer extension uses git behind the scenes, but most of the project management is done from within Slicer ( Figure 2D).

## Discussion

It should be noted that because it uses a shared, national resource (JetStream2) as the backend, there is no guarantee that at a specific time of the day, there will be sufficient resources available for everyone who would like to use MorphoCloud. In formal teaching settings like academic classes, MorphoCloud is best utilized as an asynchronous tool to supplement the curricular activities, as there is no reservation system on the JetStream2 to allow pre-reserving instances for a specific time for everyone in that class. The workshop instance types mitigate some of the issues by allowing the organizer create the instances prior to the workshop and keep them online, but these are limited to short-term events. Unfortunately for a semester long course this feature is not sufficient. For such use cases, it is best for the instructor to obtain their own JetStream2 allocation from the ACCESS, deploy their own service and customize the MorphoCloudWorkflow by changing the instance expiration time.

There are few things to emphasize. After the approval, instance creation is not automatic; as explained above, the user needs to initiate that via the /create command. Provisioning an instance from is similar to configuring a brand-new computer from scratch; it takes time. The time from issuing the /create command and the instance becoming online is at least 15 minutes and can be substantially longer. The primary factor that affects the wait time is the number of tasks in the queue that needs to happen before the system can get to the specific /create command. Given that it currently takes about 15 minutes to provision an instance, if 4-5 users issue the /create command more or less at the same time, it may take more than an hour for the last instance to be created. This is why there is a 24h grace period to the workshop. Our suggestion is the event organizer provision the instances the day before the event to have ample time for all the provisioning to take place. Also if the workshop requires additional software beyond what is provided on the MorphoCloud Instances ( Table 3), it needs to be installed by users.

**Table 3.** List of applications installed on the MorphoCloud. Additionally, users have admin rights on their own instances.

**3D Slicer** (following extensions are pre-installed, more can be installed by the user).	
**SlicerMorph:**	ImageStacks, GPA, ALPACA, PseudoLMGenerator, FastModelAlign and others
**DeCA:**	Morphometrics via dense correspondence analysis
**Photogrammetry:**	Generate textured 3D models from photographs
**MorphoDepot:**	Collaborative segmentation workflows
**MEMOs:**	AI based organ segmentation for E15 mouse embryos
**ANTsPy:**	ANTs diffeomorphic image registration and analysis library
**NNInteractive:**	AI assisted promptable interactive segmentation
**PyTorch:**	GPU accelerated tensor library for AI tools
**R and Rstudio**	
**Python3**	

MorphoCloud demonstrates that accessibility is about more than just open data; it requires open infrastructure. By abstracting away the complexity of cloud computing, we empower researchers at under-resourced institutions to participate fully in the era of big data biology.

## Software availability

- Source code available from:

https://github.com/MorphoCloud/MorphoCloudWorkflow https://github.com/MorphoCloud/MorphoCloudInstances

- Archived software available in Zenodo from these DOIs:

https://doi.org/10.5281/zenodo.18156936 (MorphoCloudWorkflow)

https://doi.org/10.5281/zenodo.18156916 (MorphoCloudInstances)

- License: BSD2

## Data Availability

This study does not rely on or generate any data.

## References

[1] Blackburn DC : Increasing the impact of vertebrate scientific collections through 3D imaging: The openVertebrate (oVert) Thematic Collections Network. *Bioscience.* Mar. 2024;74(3):169–186. 10.1093/biosci/biad120 38560620 PMC10977868

[2] Boyer DM Gunnell GF Kaufman S : MORPHOSOURCE: ARCHIVING AND SHARING 3-D DIGITAL SPECIMEN DATA. *The Paleontological Society Papers.* Sept. 2016;22:157–181. 10.1017/scs.2017.13

[3] Welcome to SlicerMorph Project. *SlicerMorph.github.io. * Accessed: Nov. 26, 2018. Reference Source

[4] Fedorov A : 3D Slicer as an image computing platform for the Quantitative Imaging Network. *Magn. Reson. Imaging.* Nov. 2012;30(9):1323–1341. 10.1016/j.mri.2012.05.001 22770690 PMC3466397

[5] Kikinis R Pieper SD Vosburgh KG : 3D Slicer: A Platform for Subject-Specific Image Analysis, Visualization, and Clinical Support. *Intraoperative Imaging and Image-Guided Therapy.* New York, NY: Springer;2014; pp.277–289. 10.1007/978-1-4614-7657-3_19

[6] Rolfe S Davis C Maga AM : Comparing semi-landmarking approaches for analyzing three-dimensional cranial morphology. *Am. J. Phys. Anthropol.* 2021;175(1):227–237. 10.1002/ajpa.24214 33483951 PMC12088985

[7] Porto A Rolfe S Maga AM : ALPACA: A fast and accurate computer vision approach for automated landmarking of three-dimensional biological structures. *Methods Ecol. Evol.* 2021;12(11):2129–2144. 10.1111/2041-210X.13689 35874971 PMC9291522

[8] Rolfe S : SlicerMorph: An open and extensible platform to retrieve, visualize and analyse 3D morphology. *Methods Ecol. Evol.* 2021;12(10):1816–1825. 10.1111/2041-210X.13669 40401087 PMC12094517

[9] Zhang C Porto A Rolfe S : Automated landmarking via multiple templates. *PLOS ONE.* Dec. 2022;17(12):e0278035. 10.1371/journal.pone.0278035 36454982 PMC9714854

[10] Zhang C Maga AM : An Open-Source Photogrammetry Workflow for Reconstructing 3D Models. *Integrative Organismal Biology.* 2023;5(1). 10.1093/iob/obad024 PMC10350669 37465202

[11] Thomas OO Zhang C Maga AM : SlicerMorph photogrammetry: An open-source photogrammetry workflow for reconstructing 3D models. *Biol. Open.* Aug. 2025;14:bio.062126. 10.1242/bio.062126 40767438 PMC12421799

[12] Rolfe SM Maga AM : DeCA: A Dense Correspondence Analysis Toolkit for Shape Analysis. *Shape in Medical Imaging.* Wachinger C Paniagua B Elhabian S , editors. Cham: Springer Nature Switzerland;2023; pp.259–270. 10.1007/978-3-031-46914-5_21

[13] Rolfe SM Mao D Maga AM : Streamlining asymmetry quantification in fetal mouse imaging: A semi-automated pipeline supported by expert guidance. *Dev. Dyn.* 2025;254(8):999–1010. 10.1002/dvdy.70028 40421888 PMC12744917

[14] Rolfe SM Whikehart SM Maga AM : Deep learning enabled multi-organ segmentation of mouse embryos. *Biol. Open.* Feb. 2023;12(2):bio059698. 10.1242/bio.059698 36802342 PMC9990908

[15] Boerner TJ Deems S Furlani TR : ACCESS: Advancing Innovation: NSF’s Advanced Cyberinfrastructure Coordination Ecosystem: Services & Support. *Practice and Experience in Advanced Research Computing 2023: Computing for the Common Good, in PEARC’23.* New York, NY, USA: Association for Computing Machinery;Sept. 2023; pp.173–176. 10.1145/3569951.3597559

[16] Hancock DY : Jetstream2: Accelerating cloud computing via Jetstream. *Practice and Experience in Advanced Research Computing 2021: Evolution Across All Dimensions, in PEARC’21.* New York, NY, USA: Association for Computing Machinery;2021. 10.1145/3437359.3465565

[17] MorphoCloud: On-Demand Cloud for 3D Slicer & SlicerMorph. *MorphoCloud: On-Demand Cloud for 3D Slicer & SlicerMorph. * Accessed: Dec. 24, 2025. Reference Source

[18] Pistorius J Martin C Sudarshan S : Exosphere - Bringing The Cloud Closer. *2020 IEEE/ACM International Workshop on Interoperability of Supercomputing and Cloud Technologies (SuperCompCloud).* Nov. 2020; pp.1–6. 10.1109/SuperCompCloud51944.2020.00006

[19] MorphoCloudWorkflow/README.md at main · MorphoCloud/MorphoCloudWorkflow. Accessed: Dec. 24, 2025. Reference Source

[20] SlicerMorphoDepot/README.md at main · MorphoCloud/SlicerMorphoDepot. Accessed: Dec. 25, 2025. Reference Source

