# Supplementary material for: MorphoCloud: Democratizing Access to High-Performance Computing for Morphological Data Analysis
Source: F1000Res. 2026 Jan 14;15:53. [Version 1] doi: 10.12688/f1000research.176328.1 (PMC13062767; doi:10.12688/f1000research.176328.1)
Supplement: Peer review history

# Reviewer report: Reviewer response for version 1

**DOI:** 10.5256/f1000research.194373.r471625

**Published:** 2026-04-08

**Authors:** Brian Metscher

**Affiliations:** 1 University of Vienna, Vienna, Vienna, Austria

The presented MorphoCloud system offers a means for anyone to use the 3D Slicer and SlicerMorph software without having to invest in new computer hardware. Slicer is a powerful and comprehensive software package with tools and add-ins for most if not all 3D image analysis tasks.

 This report is generally clear and complete, although a new user will require some level of computing knowledge to get started. The motivation and need for this software tool are clear, and the technical details are available for anyone who might need them.

 The relevant information and code are available in GitHub, which is the most-used repository for software. Users really need some familiarity with GitHub procedures, but once a user or institute has set up a MorphoCloud instance, their students or colleagues can use the Slicer software with only a web browser.

Are the conclusions about the tool and its performance adequately supported by the findings presented in the article?

Yes

Is the rationale for developing the new software tool clearly explained?

Yes

Is the description of the software tool technically sound?

Yes

Are sufficient details of the code, methods and analysis (if applicable) provided to allow replication of the software development and its use by others?

Yes

Is sufficient information provided to allow interpretation of the expected output datasets and any results generated using the tool?

Yes

Reviewer Expertise:

Evolutionary morphology, 3D imaging

I confirm that I have read this submission and believe that I have an appropriate level of expertise to confirm that it is of an acceptable scientific standard.

# Reviewer report: Reviewer response for version 1

**DOI:** 10.5256/f1000research.194373.r453831

**Published:** 2026-02-13

**Authors:** Richard Carter

**Affiliations:** 1 East Tennessee State University, Johnson City, Tennessee, USA

The authors of “MorphoCloud: Democratizing Access to High-Performance Computing for Morphological Data Analysis” present a timely solution to a problem that many PIs and students face when working with microCT datasets. That is the high compute costs associated with large three-dimensional datasets that require powerful GPU and RAM resources. While PIs may have a few such workstations in their labs, classrooms are rarely equipped with such computers. This makes instruction of these techniques prohibitive. Therefore, the solution presented here where remote access to JetStream2 (and its powerful GPUs and RAM resources) through the NSF funded ACCESS program using Morphocloud, is a game changer.

 Background information on the SlicerMorph project that runs on 3D Slicer is adequately given, including all the functionality that this module provides to biological morphologists. Furthermore, the authors highlight the open-source modular nature of Slicer where missing functionality can be added.

 As MophoCloud uses open-source projects like Github and others, the underlying code can be investigated by readers and can be adequately replicated.

 The expected results are laid out clearly in the paper. With each stage of the lifecycle described in the text and with an accompanying table or figure. As someone who has followed this exact set of steps to gain access to MorphoCloud on JetStream2, I can confidently say that these results are reproducible.

 The functionality of the software is supported and so are the limitations that authors put forward, Namely, that this system works best in workshop-like settings rather than semester long courses.

Are the conclusions about the tool and its performance adequately supported by the findings presented in the article?

Yes

Is the rationale for developing the new software tool clearly explained?

Yes

Is the description of the software tool technically sound?

Yes

Are sufficient details of the code, methods and analysis (if applicable) provided to allow replication of the software development and its use by others?

Yes

Is sufficient information provided to allow interpretation of the expected output datasets and any results generated using the tool?

Yes

Reviewer Expertise:

I am a functional morphologist that uses Slicermorph and the MorphoCloud to carry out my work on bat and dolphin head anatomy and bioacoustics. I also teach a 3D modeling course using MorphoCould and JetStream2.

I confirm that I have read this submission and believe that I have an appropriate level of expertise to confirm that it is of an acceptable scientific standard.
